# Emerging strategies of bacterial adaptation mechanisms to silver and metal oxide nanomaterials

**DOI:** 10.1093/femsre/fuaf060

**Published:** 2025-12-09

**Authors:** Lucie Suchánková, Libor Kvítek, Milan Kolář, Aleš Panáček

**Affiliations:** Department of Physical Chemistry, Faculty of Science, Palacký University Olomouc, 771 46 Olomouc, Czech Republic; Department of Physical Chemistry, Faculty of Science, Palacký University Olomouc, 771 46 Olomouc, Czech Republic; Department of Microbiology, Faculty of Medicine and Dentistry, Palacký University Olomouc, 779 00 Olomouc, Czech Republic; Department of Physical Chemistry, Faculty of Science, Palacký University Olomouc, 771 46 Olomouc, Czech Republic

**Keywords:** antimicrobial, nanomaterials, bacteria, resistance, adaptation, silver, mechanism

## Abstract

This review addresses the crucial and emerging field of bacterial adaptation to antimicrobial nanomaterials, challenging prior assumptions that their multi-level action prevents the development of reduced bacterial sensitivity. It provides a comprehensive overview of experimentally induced adaptation mechanisms across various nanomaterials (e.g. AgNPs, ZnO) and bacterial species. Bacterial adaptations encompass genetic adaptations (e.g. efflux systems, mutagenesis), biomolecule production (e.g. flagellin, exopolysaccharides forming biofilms, protein coronas), and structural changes (e.g. altered shape, cell wall thickening, enhanced motility, membrane permeability changes). The described adaptation mechanisms to nanomaterials are compared with antibiotic resistance mechanisms, emphasizing common strategies such as efflux and envelope changes, but also unique adaptations specific to nanoparticles, such as aggregation and different roles of biomolecules. The review offers insights and emerging strategies for designing safer, more effective nano-antimicrobials, including membrane potential disruption, biofilm inhibition, and size modulation. It emphasizes the need for standardized evaluation methods and future research on cross-resistance.

## Teaser

This review explores induced bacterial adaptation to antimicrobial nanomaterials, summarizing known mechanisms across nanomaterial types and bacterial species, offering insights for designing more effective and resistance-resilient nano-enabled antimicrobial strategies.

## Introduction

The introduction of antibiotics revolutionized modern medicine by dramatically reducing the extremely high mortality rates from bacterial infections, which in the pre-antibiotic era were often fatal and left many conditions, such as pneumonia, sepsis, and endocarditis, with little to no chance of survival. The introduction of penicillin and other antibiotics led to a dramatic decline in infection-related mortality, revolutionizing medical treatment, and saving countless lives. However, the initial success of antibiotics was short-lived. Resistance to penicillin began to emerge shortly after its introduction, and by the late 1940s, nearly 50% of *Staphylococcus aureus* strains isolated in the UK were already resistant. To address this, methicillin was developed, nevertheless methicillin resistance had spread globally just within 2 years of its market release (Enright et al. 2002). Unfortunately, the development of resistance has accompanied the introduction of nearly every antibiotic, including those considered drugs of “the last resort” (tigecycline, polymyxin E, daptomycin, vancomycin, linezolid) (Ventola 2015). Antibiotic resistance’s rapid and widespread emergence has now escalated into a global health crisis. The Centers for Disease Control and Prevention warns that we are already in a “post-antibiotic era,” where even “miracle antibiotics” can no longer be relied upon to treat resistant infections effectively. Antibiotic resistance has been documented in every country, leaving no region untouched. In the USA alone, ~2.8 million antibiotic-resistant infections occur annually, resulting in 35 000 deaths and significant economic losses (CDC 2019). Recent data from the 2024 WHO report further underscores the severity of the situation, revealing alarmingly high levels of resistance. Specifically, the report indicates increasing rates of resistance to third-generation cephalosporins and carbapenems in *Klebsiella pneumoniae* and widespread carbapenem resistance in *Acinetobacter* spp. across several countries, highlighting the urgent need for new strategies and interventions in combating these resistant pathogens (Sati et al. 2025).

To develop novel strategies to combat bacterial resistance, it is crucial to thoroughly understand the structure and function of bacterial cells and the mechanisms underlying resistance. The bacterial cell wall plays a critical role as the first line of defense against environmental threats, such as fluctuations in temperature, pH, and the presence of various antimicrobials. Numerous antimicrobial agents, including antibiotics (e.g. β-lactams and glycopeptides), immune-derived molecules such as lysozyme and antimicrobial peptides, specifically target components of the bacterial cell wall. Environmental stressors can damage the cell envelope and trigger a stress response aimed at maintaining cellular homeostasis under normal conditions. Therefore, preserving the integrity of the cell wall is vital for bacterial survival, as it helps the bacteria withstand the harmful effects of antimicrobials (Jordan et al. 2008, Mitchell and Silhavy 2019, Viljoen et al. 2020).

Bacteria are broadly classified into Gram-positive and Gram-negative types based on their cell wall structure, as revealed by Gram staining. Gram-positive bacteria possess a thick peptidoglycan layer enriched with teichoic and lipoteichoic acids, which provides mechanical strength and helps maintain cellular integrity. In contrast, Gram-negative bacteria feature a much thinner peptidoglycan layer enclosed by an additional outer membrane (OM), composed of lipopolysaccharides (LPS) and phospholipids. This OM functions as a robust permeability barrier, effectively limiting the entry of many antibiotics, particularly large molecules such as vancomycin and daptomycin (Silhavy et al. 2010). The selective permeability is further reinforced by porins, which allow only small hydrophilic compounds to pass through, and by efflux pumps and periplasmic enzymes like β-lactamases that degrade or expel antimicrobial agents (Mitchell and Silhavy 2019). These fundamental structural differences between Gram-positive and Gram-negative bacteria contribute significantly to their distinct susceptibility profiles and mechanisms of antibiotic resistance (Viljoen et al. 2020, Nikaido 2003, Mitchell and Silhavy 2019, Silhavy et al. 2010).

For antibacterial agents to be effective, they must achieve some effective concentration, which might be unfortunately reduced either through poor penetration of the antibiotic into the cell or by the action of efflux pumps that remove the antibiotic from the bacterial interior (Li et al. 2015). Even if some molecules penetrate this initial defense, they encounter a second line of defense in the periplasmic space (e.g. activity of β-lactamases), which provides further protection (Mitchell and Silhavy 2019). Other frequently occurring mechanisms of antibiotic resistance are linked to mutations in DNA gyrase and topoisomerase IV, modifications of antibiotic targets, and the deactivation of antibiotics through hydrolysis or other chemical alterations (Blair et al. 2015, Asif and Alvi 2018).

The escalating issue of multidrug resistance in bacteria necessitates alternative therapeutic strategies, such as identifying novel drug targets and developing innovative drug delivery mechanisms (Kirtane et al. 2021). Traditional antibiotics typically target a specific bacterial function, and currently, five primary mechanisms of antibiotic action are recognized: inhibition of bacterial cell wall synthesis, disruption of bacterial cell membranes, inhibition of protein synthesis, inhibition of nucleic acid synthesis, and inhibition of folic acid synthesis (Kohanski et al. 2010, Awad et al. 2012). However, developing entirely new antimicrobial drugs with innovative mechanisms of action is a lengthy and costly process, often leading to a lack of interest from pharmaceutical companies in this area of research. As a result, combining antibiotics with other antibacterial agents has emerged as an effective treatment strategy against resistant bacterial strains. These agents can include substances (clavulanic acid, sulbactam, tazobactam, avibactam, relebactam, vaborbactam) that reduce the production of β-lactamases, which are enzymes associated with resistance to β-lactam antibiotics (Retsema et al. 1986, Todd and Benfield 1990, Mokaddas et al. 1998).

**Figure 1.**
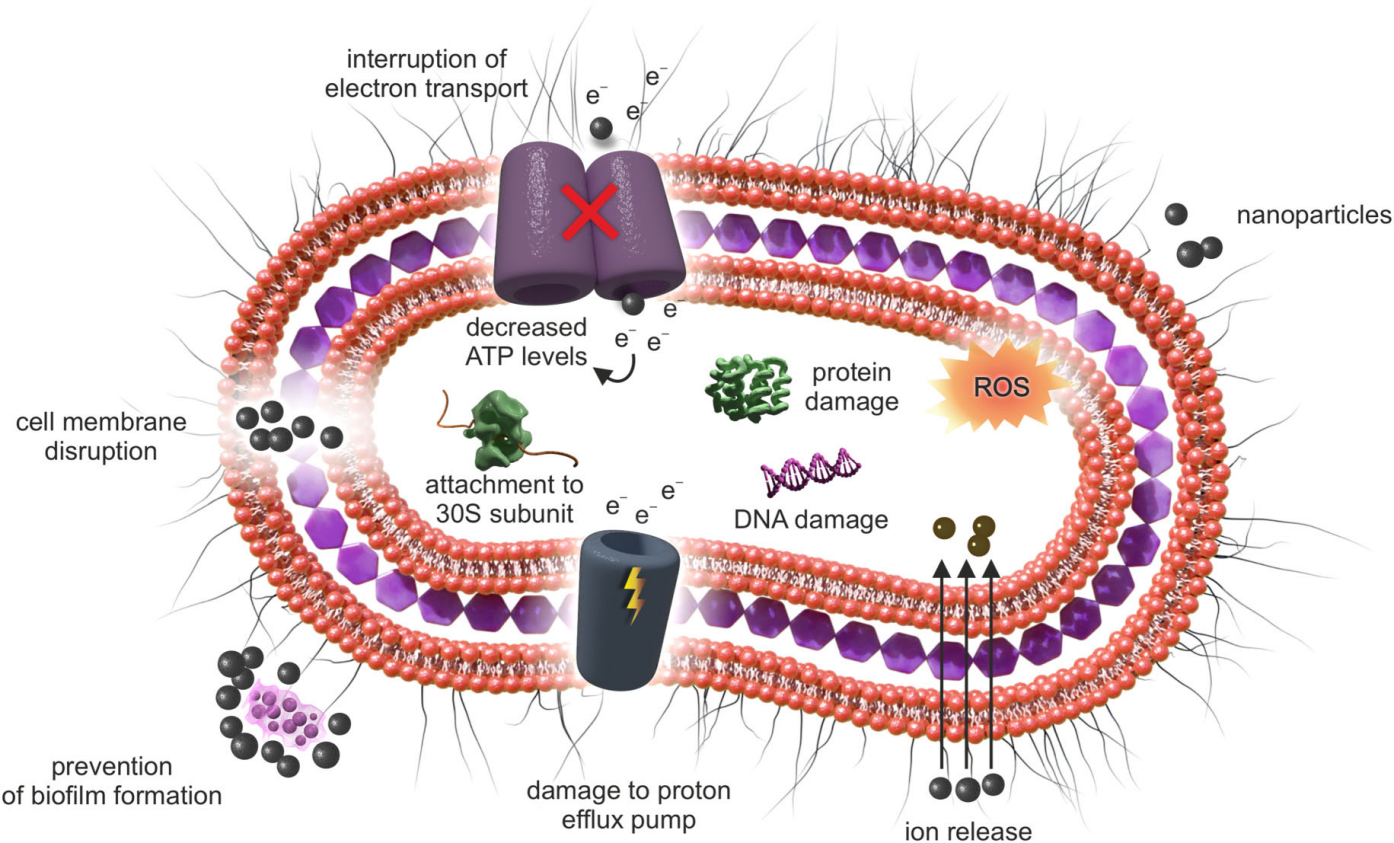
Mode of action of various nanomaterials.

Alternatively, combining antibiotics with antibacterial metal nanoparticles (MNPs) is another promising approach. Due to their high surface area-to-volume ratio and unique physical and chemical properties, MNPs exhibit non-specific antimicrobial activity and high efficacy against resistant bacteria (Wang et al. 2017). For example, silver nanoparticles (AgNPs), either used alone (Panáček et al. 2006, Kvítek et al. 2008, Suchomel et al. 2011), or combined with other antimicrobial agents (Panáček et al. 2016, Smekalova et al. 2016) demonstrate excellent antibacterial properties against a wide range of microorganisms, including multidrug-resistant strains. These findings suggest that combining an antibiotic with another nano-antimicrobial agent could produce a synergistic effect, resulting in the efficient inhibition of bacterial pathogens, including antibiotic resistant strain with significantly lower doses than using the antibiotic alone (Hochvaldová et al. 2022). Another example might be gold nanoparticles (AuNPs) (Li et al. 2014, Rao et al. 2017), zinc oxide nanoparticles (ZnO) (Kumar et al. 2017, Steffy et al. 2018), and titanium dioxide nanoparticles (TiO_2_) (Thakur et al. 2019, Dicastillo et al. 2020), which are all able to fight bacteria by various multi-level modes of action (Fig. 1) through the disruption of the cell wall and cytoplasmic membrane or via the production of reactive oxygen species (ROS), which leads to oxidative stress followed by changes in gene expression, enzymatic inhibition, and protein deactivation (Feng and Wu 2005, Wang et al. 2017, Makabenta et al. 2021).

The phenomenon of bacterial resistance has traditionally been associated with antibiotics, yet recent research demonstrates that bacteria can also adapt to alternative antimicrobial agents, including nanomaterials. Their remarkable phenotypic and genotypic plasticity, which has facilitated resistance to diverse antibiotics, likewise enables the evolution of defense mechanisms against metals and nanomaterials. Over the past 6 years, several studies have provided compelling evidence that bacteria can reduce susceptibility to nanomaterials, raising new challenges for their use in infection control. Understanding these mechanisms is therefore essential for the development of more effective and sustainable nanomaterial-based antimicrobial strategies.

## Bacterial adaptation mechanisms to nanomaterial exposure

Since bacteria have demonstrated the ability to develop resistance to antibiotics, they are also anticipated to be able to reduce susceptibility to metals and nanomaterials. Numerous studies and a limited number of reviews have provided support for this hypothesis (Kamat and Kumari 2023, Mitchell et al. 2023, Li and Xu 2024). While several previous studies have addressed bacterial responses to nanomaterials, they often focus primarily on silver-based systems and do not fully capture the complexity of adaptation mechanisms across diverse types of nanoparticles. This review systematically compares experimentally induced adaptation mechanisms across a broader spectrum of nanomaterials, including silver, zinc oxide, titanium dioxide, graphene oxide (GO), as well as gold, iron, platinum, and palladium nanoparticles. By integrating these findings with well-established antibiotic resistance strategies, the review highlights both shared mechanisms and nanoparticle-specific adaptations.

Adaptation to silver and its compounds is one of the most extensively studied forms of metal resistance in bacteria. The first identification of silver-resistant bacteria occurred in 1960 from burn wounds treated with silver nitrate (Jelenko 1969). Notable examples of bacterial strains exhibiting reduced susceptibility to silver include *Escherichia coli, Enterobacter cloacae, Klebsiella pneumoniae, Acinetobacter baumannii, Salmonella Typhimurium*, and *Pseudomonas stutzeri* (Hendry and Stewart 1979, Gupta et al. 1999, Silver 2003). Bacteria employ several mechanisms to mitigate the toxic effects of silver, while the predominant adaptation mechanism involves the efflux of Ag^+^ from the bacterial cells, (Mchugh et al. 1975, Silver 2003, Silver et al. 2006, Hobman and Crossman 2015, Li and Xu 2024, Li et al. 1997a) reducing silver cations (Ag^+^) to less toxic oxidation states has been extensively documented in previous studies and will therefore not be addressed in detail in this review (Silver 2003, Li and Xu 2024). Bacteria’s inherent ability to resist metal ions, combined with continuous genomic variations and adaptive responses to challenging conditions, suggests that bacteria may also adapt to metallic and metal oxide nanoparticles. Certain bacterial species, particularly those with adaptive traits, can also partially neutralize the toxic effects of dissolved metal cations or oxyanions, enabling them to withstand the presence of nanoparticles. By doing so, bacteria can possibly diminish not only the harmful effects of metal ions released from the nanoparticles but also other antibacterial actions of the nanoparticles. Understanding the mechanisms of adaptation to metal ions, along with resistance to antibiotics, provides crucial insights into potential adaptation mechanisms against various metallic nanomaterials. Reported survival strategy mechanisms to metallic nanoparticles are often specific, yet they also share commonalities with those observed for ionic metals and antibiotics (McNeilly et al. 2021, Li and Xu 2024b; Liu et al. 2024) and will be further discussed within this review.

### Molecular adaptation mechanisms

Bacterial adaptation mechanisms to metallic nanoparticles often originates at the molecular level, where alterations in genetic material and regulatory networks enable adaptive responses to nanoparticle-induced stress. These molecular changes include mutations in key genes, modulation of gene expression, and activation of specific regulatory pathways.

#### Efflux systems and genomic adaptations

Efflux systems and genomic adaptations constitute central strategies employed by bacteria to mitigate the cytotoxic effects of metallic nanoparticles. By actively exporting toxic metal ions and reshaping regulatory networks, bacteria reduce intracellular metal accumulation and counteract nanoparticle-induced oxidative stress.

Gunawan et al. demonstrated that Gram-positive *Bacillus subtilis* possesses a natural ability to adapt to oxidative stress induced by Ag⁺ leaching from AgNPs supported on crystalline TiO₂ during prolonged exposure. Polymerase chain reaction (PCR) analysis revealed the presence of putative cytoprotective genes: *silB* encoding a permease component of an ABC-type Ag⁺/proton antiporter, and *silP* encoding a P-type ATPase with a histidine sensor for Ag⁺ specificity. These findings indicate that *B. subtilis* harbors an intrinsic silver efflux network, which is part of an interconnected response pathway that mitigates ROS-mediated cytotoxicity (Gunawan et al. 2013).

Plasmid-encoded efflux systems also play a critical role in Gram-negative species. Losasso et al. reported reduced susceptibility to silver in *Salmonella Senftenberg* is mediated by the *SilB* gene, a key component of the tripartite *SilCBA* cation/proton antiporter. Functionally, *SilB* facilitates Ag⁺ export across the periplasm, lowering intracellular silver concentrations and mitigating toxicity (Losasso et al. 2014). Multiple studies in *E. coli* illustrate the diversity and convergence of chromosomal adaptations. Stabryla et al. showed that hypermotile *E. coli* K-12 MG1655 exposed to citrate–poly(ethylene glycol) methyl ether thiol-functionalized AgNPs (27.2 ± 4.8 nm) acquired mutations in cusS, encoding the histidine kinase sensor of the *CusS/CusR* Ag⁺–Cu²⁺ efflux system, suggesting enhanced metal ion export (Stabryla et al. 2021). Sun et al. similarly identified *cusS* mutations as a driver of enhanced Ag⁺ reflux (Sun et al. 2024). Graves et al. reported reduced susceptibility to citrate-coated AgNPs (10 nm) in *E. coli* K-12 MG1655 also through simple genomic changes, including mutations in *cusS* and *ompR*. While *cusS* mutations likely enhance CusCFBA-mediated efflux, indels in the response regulator *ompR* modify regulatory activity, facilitating survival under silver stress (Graves Jr. et al. 2015). Wu et al. extended these observations by analyzing transcriptomic and genomic changes following sublethal AgNP exposure (18 nm). Approximately 450 genes were upregulated and 462 downregulated, including suppression of quorum sensing and broad metabolic pathways, while biosynthesis of amino acid cofactors was enhanced. Recurrent mutations appeared in *cusS/cusR* (Ag⁺ efflux), *ompR* (membrane permeability), and *arcA/arcB* (energy metabolism), indicating an integrated response linking efflux, regulatory adaptation, and metabolic adjustment (Wu et al. 2022). Faghihzadeh et al. reported additional simple genomic changes in *E. coli* K-12 MG1655, including mutations in *copA* and *cusA*, emphasizing efflux and the fact that culture growth conditions can significantly influence the trajectory of bacterial adaptations (Faghihzadeh et al. 2018). Finally, Kędziora et al. observed loss-of-function mutations in *cusS* and *ompR* in *E. coli* ATCC 11229 S2 and in *cusS* or *tamB* in *K. pneumoniae* ATCC 4352 S1 after prolonged AgNP exposure, correlating reduced susceptibility (Kędziora et al. 2020).

Collectively, these studies reveal a convergent strategy among diverse bacterial species: mutations in efflux-related genes (*cusS, cusR, ompR, silB, silP, copA, cusA, tamB*) enhance metal ion export, regulate membrane permeability, and mitigate oxidative stress. Moreover, these adaptations often involve broader regulatory and metabolic adjustments, demonstrating the interplay between adaptation, survival, and cellular fitness under nanoparticle-induced stress. The repeated identification of similar genetic changes across independent studies highlights the evolutionary robustness of efflux-mediated adaptation as a core mechanism for bacterial adaptation to metallic nanoparticles.

#### Regulation of gene expression and metabolism

Bacterial adaptation to metallic nanoparticles often involves complex regulatory adjustments at the gene expression level that lead to significant alterations in metabolic pathways, which enhance survival under long-term metal-induced stress. Exposure triggers modulation of specific genes and pathways that eliminate ROS, repair DNA damage, and hydrolyze misfolded or improperly assembled proteins (Poirier et al. 2013). Biofilm-forming bacteria may synthesize cysteine-rich proteins or metal-binding peptides to sequester silver ions, mitigating toxicity and supporting biofilm development (Huang et al. 2018). Valentin et al. reported that mutations in cysteine metabolism genes were identified in *S. aureus* ATCC 25923 strains resistant to AgNPs supported on TiO₂ as well as to ionic silver. Specifically, mutations in the l-cystine binding protein *TcyA* (AgNP-resistant) and the operon repressor *CymR* (ionic silver-resistant) likely minimize cysteine-mediated redox imbalance, reducing extracellular cysteine influx and lowering intracellular cysteine pools. These adaptations illustrate how bacteria can fine-tune amino acid metabolism to reduce metal-induced oxidative stress (Valentin et al. 2020).

Metabolic pathway adaptations complement these regulatory responses. Long-term serial passaging led to a single-nucleotide variation in *purR* (TCT→TAT), predicted to decrease its DNA-binding affinity and derepress the purine biosynthesis genes. This enhanced nucleotide availability may support DNA replication and repair, counteracting AgNP-induced DNA and protein damage, and was not observed in ionic silver-resistant strains, suggesting nanoparticle-specific stress adaptation (Valentin et al. 2020). Radical-mediated mutagenesis in *E. coli* K12,where ROS lead to DNA damage and subsequent mutations, under Al₂O₃ and ZnO nanoparticle exposure produced recurrent mutations in *gyrA, marR*, and *rcsC*, with Al₂O₃ uniquely affecting *soxR* and *rob*, and ZnO targeting *gyrB, mazG*, and *acrR*, demonstrating both shared and metal-specific genomic adaptations (Zhang et al. 2018b). Similarly, sub-inhibitory GO exposure in *E. coli* K12 MG1655 persistently modulated the Cpx envelope stress response (*cpxAR–nlpE–cpxP*), reduced periplasmic stress, and increased secretion of proteases (*DegP, Clp, Lon, FtsH, HslVU*). GO-exposed cells formed approximately twice the biofilm biomass of ancestor cells and overexpressed curli (*csgB*), motility (*motB*), adhesin (*ypjA*), and quorum-sensing regulator (*lsrR*) genes, illustrating integrated regulation of stress response, biofilm formation, and protein quality control (Zhang and Zhang 2020a).

Interestingly, the study by Du et al. on *Listeria monocytogenes* revealed a unique adaptive strategy in response to prolonged AgNP exposure. The resistant strain exhibited marked downregulation of virulence-related genes *(hly, prfA, plcA, plcB, inlA, inlB, zmp*), which was corroborated by reduced hemolytic activity. While bacterial exposure to stressors or nanomaterials often leads to enhanced pathogenic traits, in this case, adaptation was associated with a suppression of virulence. This observation suggests a potential trade-off mechanism, whereby bacteria prioritize survival under nanoparticle stress over maintaining pathogenic potential, adding a novel dimension to our understanding of nanomaterial-induced adaptations (Du et al. 2025).

Metal-specific regulatory and transport responses further enhance bacterial adaptation. Guo et al. observed that *P. aeruginosa* PAO1 exposed to sublethal CuO NPs upregulated genes coding for copper adaptation, particularly in the Pf1 bacteriophage cluster, promoting biofilm development and enhancing the efflux of CuO NPs and Cu ions, thereby reducing intracellular toxicity (Guo et al. 2017). Overall, almost all adaptation mechanisms to metallic nanoparticles involve genetic changes, with some being thoroughly explored in articles such as Li et al (2019). However, some studies, including Panáček et al., detected no mutations, and in other studies, genetic changes were not investigated at all (Zhang et al. 2018a, Elbehiry et al. 2019, Ellis et al. 2019). Since most adaptation mechanisms are encoded by several genes that can be transferred between bacteria, the possibility of genetic transfer and cross-resistance to other MNPs, their metal ions, or antibiotics should be thoroughly studied. So far, only (Elbehiry et al. 2019, Cui et al. 2020) have studied cross-resistance between AgNPs and various antibiotics (with negative results), and just two other studies have confirmed cross-resistance to silver ions (Ellis et al. 2019, Cui et al. 2020, Valentin et al. 2020).

These molecular and genetic adaptations often manifest through the production of various biomolecules and structural changes, which are further explored in the following section.

### Biomolecular production and extracellular mechanisms

Building upon molecular adaptations that involve alterations in genetic material and regulatory networks, bacteria deploy a sophisticated and dynamically regulated arsenal of extracellular biomolecules and structural adaptations to counteract the toxic effects of nanoparticles. These defenses, which are a form of biomolecule production, range from immediate physical shielding by components like the cell wall, plasma membrane, and capsules that adsorb and block metal ions, to the production of complex extracellular polymeric substances (EPS) and the formation of protein coronas. Together, these mechanisms form an integrated extracellular defense system that complements intracellular adaptations and is crucial for long-term bacterial survival under nanoparticle-induced stress (Ferris and Beveridge 1984, El-Helow et al. 2000, Taniguchi et al. 2000, Bonnefoy and Holmes 2012).

#### Extracellular barriers, biofilm, and quorum sensing

Bacteria employ a range of extracellular strategies to mitigate nanoparticle toxicity, creating both physical and chemical barriers. Structural components such as the cell wall, plasma membrane, and capsules adsorb and block metal ions, while prolonged exposure promotes their accumulation in the extracellular matrix through adsorption, complexation, ion exchange, and precipitation, thereby limiting penetration into the periplasm and cytoplasm (Teitzel and Parsek 2003, Pereira et al. 2011, Mahto et al. 2022). In parallel, bacteria secrete EPS composed of polysaccharides, proteins, lipids, and nucleic acids. EPS physically traps nanoparticles, reduces their effective surface area, inhibits dissolution, and facilitates processes such as silver sulfidation, collectively lowering toxicity. Together, these mechanisms form an integrated extracellular defense system that acts in concert with intracellular adaptations (Davies 2003, Flemming and Wingender 2010, Kang et al. 2014, Yang et al. 2022).

**Figure 2.**
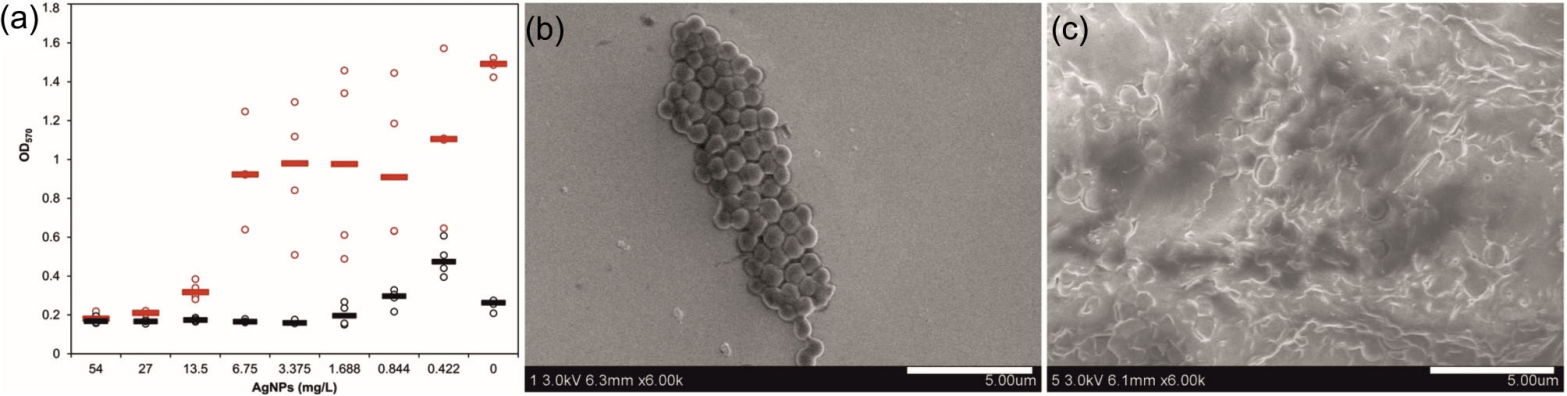
Graph showing the optical density of biofilm stained by CV, formed in culture media containing different amounts of AgNPs in the presence of susceptible (black) and resistant (red) *S. aureus*. SEM images of susceptible (b), resistant (c) *S. aureus* treated with sub-inhibition concentration of silver nanoparticles. Reproduced with permission from Hochvaldová et al., Communications Biology, 2024, © Springer Nature.

Molecular and structural adaptations of EPS under prolonged exposure have been well documented. Khan et al. observed that sawage isolates *Bacillus pumilus* treated with AgNPs exhibited similar growth kinetics to those without NPs. They suggested that the capping of nanoparticles by bacterially produced exopolysaccharides was the probable mechanism of adaptation because the bacterial exopolysaccharide-capped silver NPs showed less activity (Khan et al. 2011). Similar nanoparticle entrapment functions of the exopolysaccharides were described in *Enterococcus faecali*s ATCC 29212 (Cui et al. 2020) and *S. aureus* 008. In our recent work, *S. aureus* resistant strains exhibited significantly higher biofilm levels than susceptible strains (Fig. 2), (Hochvaldová et al. 2024). Faghihzadeh et al. examined the kinetic, metabolic and macromolecular response of *E. coli* K-12 MG1655 to chronic nanoparticle exposure in continuous culture in bioreactors. In the AgNPs treated cultures, the proportion of β-sheet structures in EPS proteins was significantly higher than that of α-helix structures (β/α ratio > 2), as determined by ATR-FTIR spectroscopy. This shift in secondary structure suggests the formation of a protein corona, as proteins typically undergo conformational changes upon adsorption onto nanoparticle surfaces. Specifically, α-helical regions being more compact and ordered, may unfold and reorganize into β-sheet-rich configurations when interacting with the nanoparticle surface, reflecting increased conformational entropy. This structural transformation, along with supporting evidence from Cryo-TEM and thermogravimetric analysis, confirms that AgNPs were coated with extracellular proteins, forming a biologically derived protein corona (Faghihzadeh et al. 2018).

Long-term adaptation to other nanomaterials, such as GO, further demonstrates the plasticity of extracellular defense. Zhang et al. showed that in *E. coli* K12 MG1655, long-term GO exposure doubled biofilm biomass and induced overexpression of curli (*csgB*), motility (*motB*), adhesin (*ypjA*), and quorum-sensing regulator (*lsrR*) genes, highlighting the integrated regulation of extracellular and behavioral adaptations (Zhang and Zhang 2020a).

Quorum sensing as a regulatory mechanism of biofilm adaptation is central to long-term adaptation. In *P. aeruginosa* PAO1, sublethal concentrations of 10 nm polyvinylpyrrolidone-coated AgNPs promoted biofilm formation through upregulation of QS genes (*lasR, lasI*), LPS biosynthesis genes (*sagS, pslA*), and the multidrug efflux gene *mexA*, accompanied by increases in EPS protein (+114 ± 32%) and sugar (+55 ± 3%) content (Yang and Alvarez 2015). Wu et al. reported that sublethal AgNP exposure in *E. coli* MG1655 suppressed quorum sensing pathways while broadly affecting other metabolic processes. Although genome-wide mutation rates remained unchanged, six QS-related genes accumulated mutations, including *oppF, pdeR, ydcZ, SdiA, rcsA*, and *ftsY*, linking QS modulation directly to the increased adaptation at the population level (Wu et al. 2022). Similarly, Li et al. reported that reduced susceptibility to noble metal-based nanomaterials (Pt, Pd) in *P. aeruginosa* involved upregulation of *lasR*, which reduced intracellular ROS and enhanced biofilm formation. Knocking out *lasR* impaired biofilm development and increased ROS accumulation, confirming the QS-dependent adaptation mechanism (Li et al. 2021).

**Figure 3.**
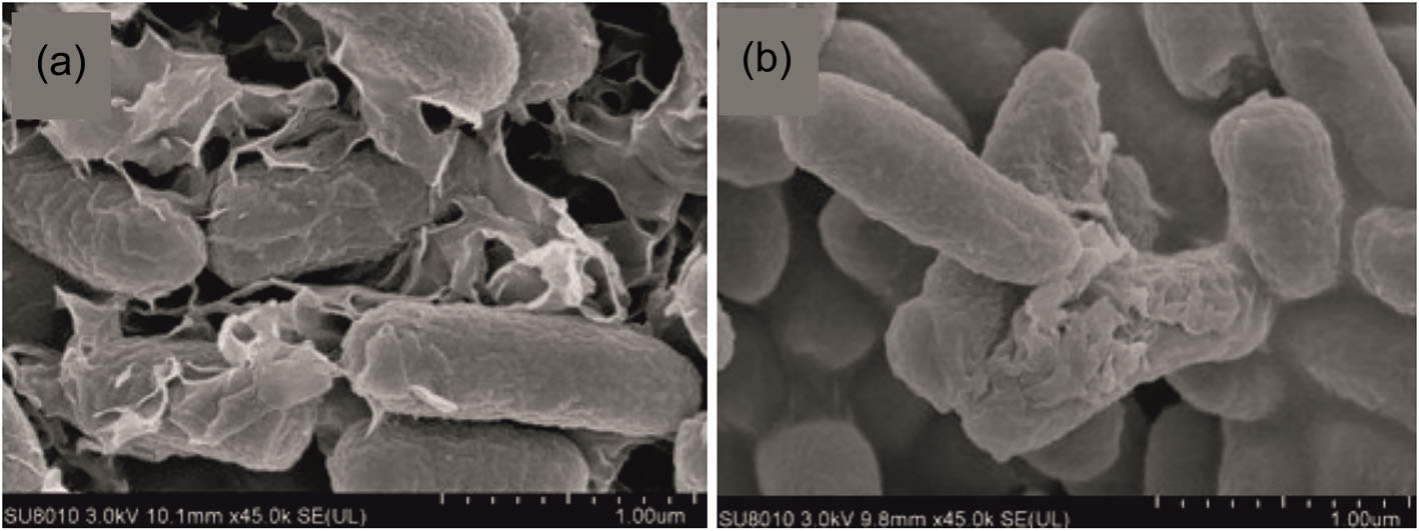
SEM images of the original (left) and the evolved (right) strain from day 7, treated with 20 mg/l GO, while evolved cells bind to GO less. Reproduced with permission from Zhang et al., Environmental Science: Nano, 2018, © Royal Society of Chemistry.

Long-term adaptation to non-lethal GO further exemplifies QS-mediated adaptation. Initially, GO adsorbs key QS signals (N-3-oxo-dodecanoyl homoserine lactone) and QS-regulated proteases *in P. aeruginosa* PAO1, reducing biofilm formation without altering QS gene transcription (*lasI, lasR, rhlI, rhlR*). Over ∼160–200 generations, bacterial cells adapt by overproducing QS signals and proteases, coupled with alterations in cell surface properties that reduce GO adsorption (Fig. 3). This combination restores QS functionality, biofilm formation, and reduced susceptibility to GO (Zhang et al. 2018c). These adaptations show that bacteria can remodel their QS systems under prolonged nanoparticle stress, coordinating extracellular defenses, biofilm formation, and intercellular communication to enhance survival.

#### Pigment production

Bacterial pigment production represents an additional protective strategy against nanoparticle-induced stress, as certain pigments can chemically interact with metal ions, modulating their reactivity and toxicity. Ellis et al. observed that *P. aeruginosa* could develop stable adaptation to citrate-stabilized AgNPs accompanied by distinct phenotypic changes, including alterations in pigment production, from yellow-green to blue–green, indicative of modifications in phenazine metabolism. The 715npRPs variant produced blue–green pyocyanin from early passages, a redox-active phenazine capable of reducing Ag⁺ to Ag⁰, potentially limiting silver bioavailability. The 718npRPs variant maintained yellow-green pigments, likely representing siderophores such as pyochelin and pyoverdin. Pyochelin binds silver ions and could facilitate internalization, while pyoverdin may act protectively by preventing Ag⁺ uptake. These pigment alterations suggest that phenazine-mediated modulation of silver bioavailability contributes to defense mechanism (Ellis et al. 2019).

#### Flagellin and motility

Bacteria employ dynamic phenotypic adaptations to resist nanoparticle toxicity, including flagellin production, which can reduce antimicrobial effectiveness without necessarily involving genetic changes. Flagellin, the structural protein of bacterial flagella, mediates the aggregation and destabilization of nanoparticles, forming part of an integrated defense that can complement both extracellular and intracellular mechanisms.

**Figure 4.**
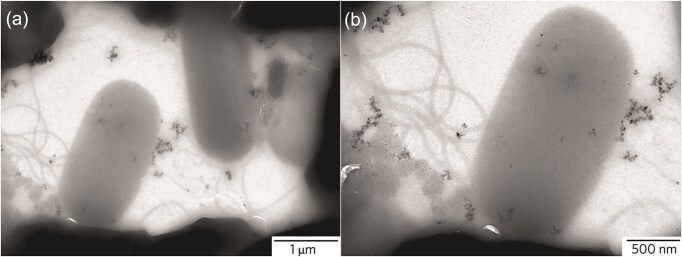
Aggregation of silver NPs induced by resistant *E. coli*. Panels (a) and (b) show TEM images at different magnifications to illustrate the aggregation behavior. Reproduced with permission from Panáček et al., Nature Nanotechnology, 2018, © Springer Nature.

In Gram-negative bacteria such as *E. coli* and *P. aeruginosa*, adhesive flagellin proteins induce aggregation and destabilization of AgNPs (Fig. 4), thereby reducing their antibacterial activity. This process occurs without genetic alteration and cannot be overcome by additional nanoparticle stabilization. However, adaptation mediated by flagellin can be counteracted by inhibiting its production, for instance, using pomegranate rind extract (Panáček et al. 2018). This flagellin-dependent mechanism is often a part of a broader adaptation strategy, as aggregation can also involve bacterial pili, OM proteins, ribosomal proteins, or enzymes (Stabryla et al. 2021). Furthermore, flagellin-mediated defense can operate in combination with other mechanisms, such as a biphasic adaptation strategy in *E. coli*, where initial nanoparticle precipitation by flagellin overexpression is followed by activation of the *Cus* efflux pump to expel silver ions, thus combining extracellular aggregation with ionic efflux (Sun et al. 2024). Similarly, Alhajar et al. observed that AgNPs-resistant *E. coli* exhibited reduced biomass yield, lower ROS generation, and reliance on efflux pumps for survival, with whole-genome sequencing revealing mutations in genes related to OM proteins, lipid A biosynthesis, transporters, motility structures, and the *marR* regulator, pointing to membrane remodeling and oxidative stress tolerance as complementary adaptation strategy (Alhajjar et al. 2022).

Chronic nanoparticle exposure can also remodel bacterial motility. Research by Du et al. with *L. monocytogenes* demonstrates that dynamic motility strategies can play a critical role in adaptation to nanoparticles. The resistant strain exhibited enhanced gliding motility, accompanied by upregulation of genes involved in chemotaxis and two-component regulatory systems, indicating an active behavioral response to AgNP stress. Unlike classical flagellin-mediated aggregation or flagellar motility observed in other bacteria, this enhanced gliding represents a distinct mode of movement, enabling the bacteria to spatially evade nanoparticle exposure (Du et al. 2025). Zhang et al. reported that long-term adaptation of *E. coli* K12 MG1655 to nanostructured TiO₂ significantly increased swimming ability (∼66% increase), enhanced path speed (7.49 μm s⁻¹ vs. 4.36 μm s⁻¹), and produced extended flagella with denser fimbriae. These changes correlated with upregulation of genes involved in flagellar assembly, fimbriae biosynthesis, and chemotaxis, suggesting a strong link between nanoparticle stress and motility-driven defense (Zhang et al. 2020). In contrast, long-term exposure to thiol-capped AuNPs produced the opposite phenotype: resistant *E. coli* strains lost flagella and displayed reduced swarming motility, accompanied by extensive surface remodeling. These findings highlight the versatility of motility-related adaptations, which may either enhance or suppress flagellar expression depending on the type of nanoparticle stress encountered (Zheng et al. 2021).

### Structural and morphological adaptations

Phenotypic adaptation to nanoparticles often involves observable physical changes in bacterial cells. These structural and morphological adaptations include alterations in cell shape and size, thickening of the cell wall, and modifications to membrane composition. Such changes can significantly affect how nanoparticles interact with bacterial cells, reducing nanoparticle penetration or toxicity, and thus contribute to bacterial survival under nanoparticle-induced stress. This section reviews these adaptive modifications and their implications for bacterial survival against nanoparticles.

#### Membrane changes

Alterations in the bacterial cell membrane constitute a key adaptive strategy to mitigate nanoparticle toxicity. By modulating membrane fluidity, permeability, and surface composition, bacteria reduce nanoparticle penetration, limit intracellular interactions, and maintain cellular homeostasis. Central mechanisms include fatty acid isomerization, stress-induced envelope remodeling, and changes in surface molecules such as LPS, which collectively enhance survival under prolonged nanoparticle exposure.

Exposure to metallic nanoparticles and GO triggers dynamic remodeling of membrane lipids. For example, in *P. putida* mt-2, repeated sublethal silver exposure induced a marked increase in the trans/cis ratio (from 0.1 to 0.85) of unsaturated fatty acids, reflecting an adjustment to maintain membrane integrity, while overall surface hydrophobicity (contact angle 30–40°) remained unchanged, indicating no alteration of surface properties (Hachicho et al. 2014). Similar patterns were observed in *P. putida* F1 exposed to nanoscale zero-valent iron (nZVI), where persistent cells displayed increased membrane rigidity, a more negative surface potential, and a higher saturated/unsaturated fatty acid ratio. Acute exposure of native cells triggered rapid cis–trans isomerization of fatty acids, whereas pre-adapted cells showed attenuated isomerization, indicating that repeated exposure selects for phenotypic variants with preconditioned membrane composition that limits nanoparticle-cell interaction (Kotchaplai et al. 2017).

Membrane remodeling can also involve genetic adaptation. In *P. putida* KT2440, whole-genome sequencing after AgNP (12.4 ± 4 nm, uncoated) exposure revealed mutations in genes encoding cell surface components (*ftsZ, envZ, gacS, PP_2758*), whereas Ag⁺ exposure primarily affected cytoplasmic proteins (tauB-II, thiL, PP_2397), with *copA* mutations shared between both treatments. These findings suggest that both ionic and particulate silver exert selective pressure on membrane-associated genes, contributing to long-term adaptation (Dong et al. 2022). Similarly, long-term exposure of *E. coli* K12 MG1655 to sub-inhibitory GO induced extensive membrane remodeling. Lipid composition shifted toward lower saturated/unsaturated fatty acid ratios, driven by increased palmitoleic acid, which increased fluidity and permeability. LPS content changed by >25%, accompanied by upregulation of biosynthetic genes (*lpxBCDK, arnT*). Metabolically, GO-exposed cells shifted from the TCA cycle toward glycolysis and the pentose phosphate pathway, accumulated lactate, experienced intracellular acidification (pH 5–6 vs. 7.1), and showed elevated K⁺/Na⁺ ratios (>6-fold). Acid tolerance genes (*hdeB, cadAB*) were enriched, enabling growth under acidic stress, while functionally, these cells displayed increased adhesion, invasion into macrophages, and stronger pro-inflammatory responses (Zhang and Zhang 2020a).

Overall, these studies highlight that bacterial membrane adaptations to nanoparticles integrate structural, biochemical, and sometimes genetic changes. Fatty acid remodeling, LPS modification, and surface charge adjustment work in concert to reduce nanoparticle-cell interactions, enhance stress tolerance, and promote survival under prolonged nanoparticle exposure, demonstrating the central role of membrane plasticity in bacterial adaptation against MNPs.

#### Changes in shape and cell wall

Bacterial adaptation to nanoparticle-induced stress frequently involves remodeling of cell morphology and cell wall architecture. Such alterations can influence the surface-to-volume ratio, mechanical strength, and permeability of the bacterial envelope, thereby modulating nanoparticle uptake and reducing toxicity. Reported structural changes include filamentation, cell wall thickening, shape transformation, flagellar loss, and size reduction, all of which contribute to enhanced tolerance.

**Figure 5.**
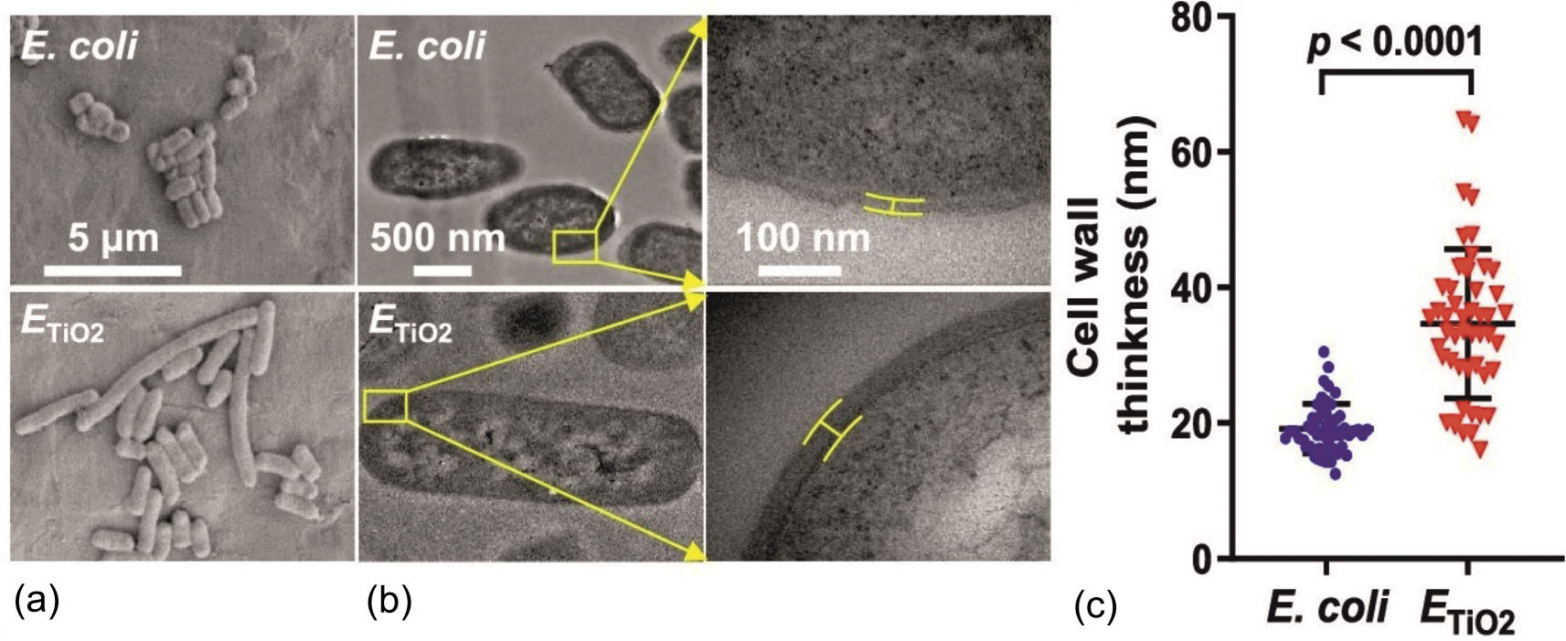
Morphogenesis of *E. coli* with and without exposure by TiO_2_ NPs exposure (a) SEM images showing the elongation of ETiO_2_, (b) TEM images indicating the thickening of the cell wall in ETiO_2_, (c) Quantification of the cell wall thickness based on TEM images. Reproduced with permission from Zhang et al., Environmental Science & Technology, 2020, © American Chemical Society.

**Figure 6.**
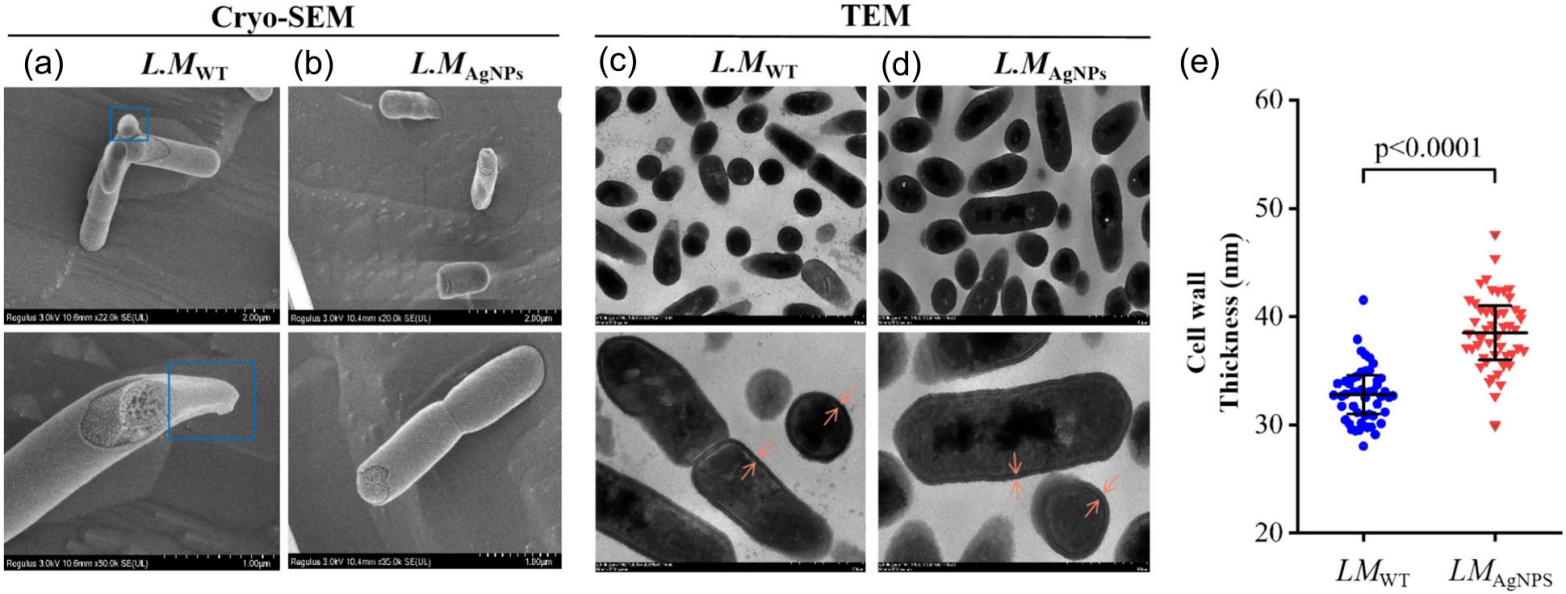
Bacterial section Cryo-SEM images (a, b) of bacterial cell wall and TEM images (c, d) indicating the thickening of the cell wall in *L. monocytogenes* (a, c) and bacteria treated with AgNPs (b, d). Measurement of the cell wall thickness based on 50 randomly selected cells (e). Reproduced with permission from Du et al., International Journal of Food Microbiology, 2025, © Elsevier.

One frequently reported adaptation is cell elongation and cell wall thickening. For instance, chronic exposure of *E. coli* K12 MG1655 to nanostructured TiO₂ resulted in filamentous phenotypes with cell walls ∼80% thicker than those of untreated cells, along with enhanced tolerance to oxidative stress and increased biofilm formation. These changes suggest that reinforcement of the bacterial surface and altered growth modes can mitigate nanoparticle stress. As shown in Fig. 5, some rod-shaped cells became abnormally elongated, and their cell walls thickened after exposure to TiO_2_ NPs (Zhang et al. 2020). The *L. monocytogenes* strain provides a clear illustration of similar adaptations in a Gram-positive organism. Detailed TEM analyses (Fig. 6) revealed sharp-tapered poles and a 17.4% increase in cell wall thickness compared to the wild-type strain. The thickened cell wall and altered cell shape likely reduce nanoparticle penetration and enhance cellular protection, directly exemplifying the broader mechanisms of cell wall thickening and shape transformation observed across diverse bacterial species (Du et al. 2025).

**Figure 7.**
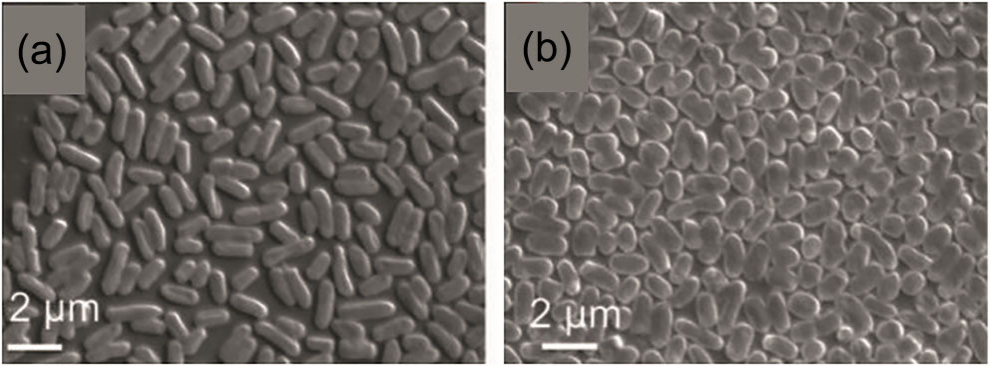
Scanning electron microscopy images of the changing shape of *E. coli* grown on a medium without (left) and with ZnO NPs (right). Reproduced with permission from Zhang et al., Advanced Biosystems, 2018, © Wiley-VCH.

**Figure 8.**
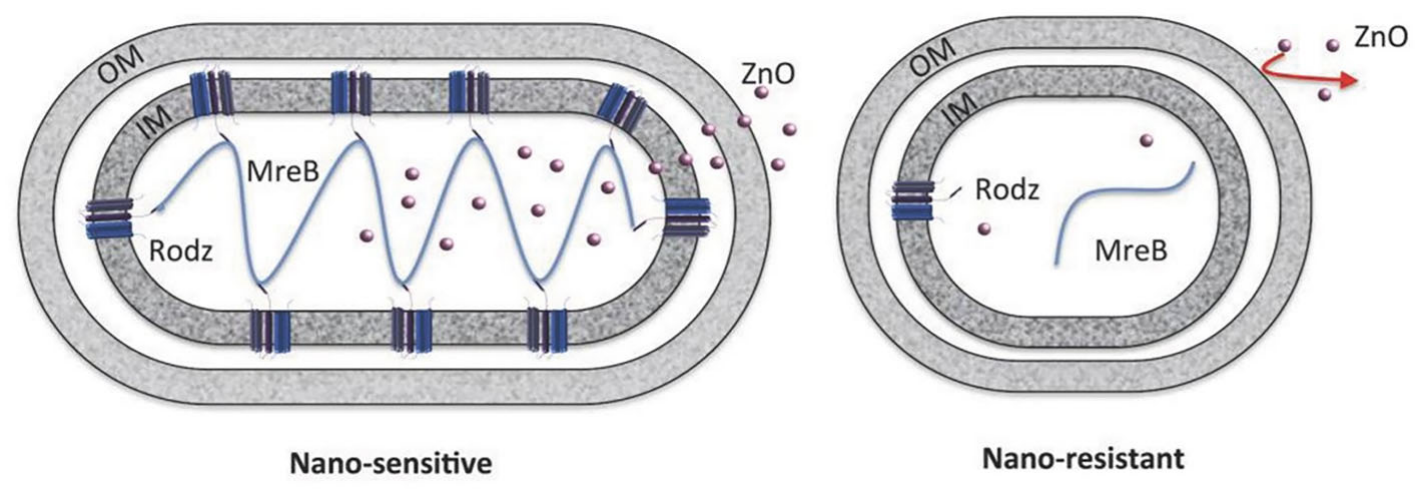
Schematic drawing of the structure of susceptible (left) and resistant (right) *E. coli* after exposure to ZnO NPs. Reproduced with permission from Zhang et al., Advanced Biosystems, 2018, © Wiley-VCH.

Another common adaptation is shape transformation and was described in *E. coli* ATCC 8739 exposed to 18 nm ZnO nanoparticles. Cells shifted from rod-like to oval morphologies (Fig. 7), lowering their length-to-width ratio and surface-to-volume ratio. This was associated with interference in the *RodZ*–*MreB* system that maintains rod shape, as well as possible changes in porin (*OmpC/OmpF*) and efflux pump expression. By minimizing the effective surface exposed to nanoparticles, these oval-shaped variants could limit ZnO penetration and toxicity. When ZnO diffused into the bacteria, it could bind to *RodZ*, thereby blocking the interaction with *MreB*, which resulted in oval-shaped, resistant *E. coli* with a lower surface-to-volume ratio, potentially impeding the diffusion of ZnO NPs into the bacterium (Fig. 8) (Zhang et al. 2018a).

**Figure 9.**
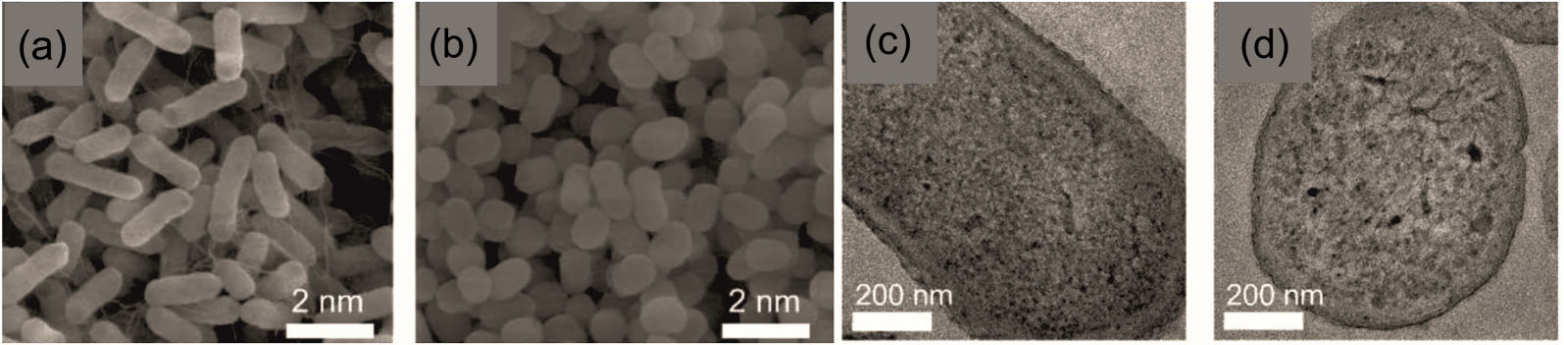
SEM and TEM images of (G, I) *E. coli* and (H, J) *E. coli* after prolonged exposure to AuNPs, respectively. Reproduced with permission from Zheng et al., Nano Letters, 2021, © American Chemical Society.

In addition, extensive surface remodeling has been observed during long-term adaptation to 4,6-diamino-2-pyrimidine thiol-capped gold nanoparticles (AuDAPTs). Prolonged exposure of *E. coli* to AuDAPTs led to a smaller cell size (Fig. 9), loss of flagella (a contrasting adaptation to the enhanced flagellar expression observed with other nanomaterials), reduced swarming motility, and changes in membrane permeability and LPS composition. Interestingly, these resistant strains bound nanoparticles similarly to wild-type cells, but maintained structural integrity upon binding, highlighting that protection strategy arose from surface modifications rather than altered electrostatics. Such remodeling was also nanoparticle size-specific, as adaptive mechanism to one particle size did not confer cross-resistance to others (Zheng et al. 2021).

## Discussion

The study of bacterial adaptations induced by prolonged exposure to sublethal concentrations of MNPs has gained momentum since 2018, particularly following the pioneering work of Panáček et al., which focused on reduced susceptibility to AgNPs. This work has significantly accelerated research in this area. Before this, only a few studies had explored various mechanisms by which bacteria withstand the adverse conditions caused by the presence of MNPs, such as AgNPs and CuO NPs. To the best of our knowledge, no comprehensive review has yet been published that fully describes adaptation mechanisms induced by various nanomaterials. While some reviews include brief sections on this topic within the broader context of antibacterial nanomaterials, others have primarily concentrated on silver nanoparticles or silver ions, often offering a limited view of the diverse range of adaptation mechanisms (Silver 2003, McNeilly et al. 2021, Li and Xu 2024, Liu et al. 2024). Building on this gap, the present review aims to provide a structured overview of the diverse mechanisms by which bacteria adapt to nanomaterial exposure. These strategies range from molecular responses, such as efflux pump activation, genomic alterations, and regulation of gene expression and metabolism, to extracellular defenses including barrier formation (cell wall, plasma membrane, and capsules), sequestration of metal ions through EPS, biofilm formation, pigment production, and motility-related changes, as well as structural adaptations involving modifications of membrane composition, cell shape, and wall thickness. In the following sections, these mechanisms will be systematically examined across different bacterial types and classes of nanomaterials. Furthermore, parallels and distinctions with antibiotic resistance will be highlighted, and particular attention will be given to experimental approaches used to demonstrate adaptation mechanisms induction by nanoparticles. Finally, potential interventions to counteract or mitigate newly built nanoparticle survival strategy will be considered, providing a comprehensive framework for understanding both the challenges and opportunities in this emerging field.

### Adaptation mechanisms across bacterial types and nanomaterials

The emergence of bacterial adaptation mechanisms to nanomaterials has been most extensively studied for AgNPs, but similar adaptive responses have been described for other nanostructures, including ZnO (Zhang et al. 2018a, b), TiO_2_ (Zhang et al. 2020), Al_2_O_3_ (Zhang et al. 2018b), ZVI (Kotchaplai et al. 2017), and GO (Zhang et al. 2018c, 2020a) nanomaterials (Table 1). While Gram-negative bacteria such as *E. coli* and *P. aeruginosa* have been the primary focus, Gram-positive species have been able to adapt, although their mechanisms remain less well explored. Across bacterial types, certain strategies appear to be universal, most notably the activation of efflux systems (Zhang et al. 2018b, Gunawan et al. 2013, Graves Jr. et al. 2015, Faghihzadeh et al. 2018, Valentin et al. 2020) that lower intracellular nanoparticle or ion accumulation, the formation of extracellular barriers such as biofilms and exopolysaccharide matrices (Elbehiry et al. 2019, Cui et al. 2020, Kędziora et al. 2020, Valentin et al. 2020, Hochvaldová et al. 2024), and phenotypic changes as cell wall thickening (Zhang et al. 2020, Du et al. 2025), shape alternation (Zhang et al. 2018a), bacterial filamentation.

**Table 1.** Mechanisms of resistance to various nanomaterials described in the literature up to date.

NPs	bacteria	Gram	Passage	Step	Gene	Cross	Stable	Reversed	Mechanism	Author
Ag	*E. coli*	-	225	100	Y	-	-	-	Efflux of silver ions mediated by (mdtb gene)	(Graves Jr. et al. 2015)
Ag	*E. coli*	-	17	17	Y	-	Y	-	Overexpression of efflux-related genes (copA, cusA), lipid biosynthesis regulation, and upregulation of cpsB and fabR associated with extracellular β-sheet-rich protein corona formation	(Faghihzadeh et al. 2018)
Ag	*E. coli, P. aeruginosa*	-	20	9	N	-	Y	Y—PGRE	Overexpression of flagellin leading to enhanced aggregation	(Panáček et al. 2018)
Ag	*E. coli*	-	-	-	Y	-	-	-	Activation of Suf system and cysteine biosynthesis, increased efflux activity(cuS), downregulation of porin synthesis genes	(Pareek et al. 2022)
Ag	*E. coli*	-	24	24	Y	-	N	-	Upregulation of cusS/cusR efflux regulators and ompR involved in osmoregulation	(Wu et al. 2022)
Ag	*E. coli*	-	10	10	Y	-	-	-	Overexpression of flagellin resulting in enhanced motility	(Alhajjar et al. 2022)
Ag	*E. coli*	-	14	14	Y	-	-	Y—CCCP	Flagellin overproduction combined with activation of the CusCFBA efflux system	(Sun et al. 2024)
Ag (Ag_2_S)	*E. coli*	-	100	100	Y	-	-	-	Altered energy metabolism, oxidative stress response, Cu efflux activation, and induction of DNA repair/SOS response pathways	(Li et al. 2019)
Ag, Ag+	*E. coli*	-	20	8	Y	-	Y	-	Mutation in cusS regulatory gene increasing silver ion efflux	(Stabryla et al. 2021)
Ag, CNGs	*E. coli, S. aureus*	-,+	20	-	N	-	-	-	Not described	(Mao et al. 2022)
Ag	*L. monocytogenes*	+	35	28	Y	-	Y	-	Cell wall thickening, biofilm enhancement, and altered motility	(Du et al. 2025)
Ag	*P. aeruginosa*	-	9	3	-	Y -AgNO_3_	Y	-	Increased production of phenazine pigments involved in redox cycling and AgNP detoxification	(Ellis et al. 2019)
Ag	*P. aeruginosa*	-	-	1	Y	-	-	-	Enhanced biofilm formation and upregulation of quorum sensing and mexA efflux pump genes	(Yang and Alvarez 2015)
Ag, Ag^+^	*P. putida*	-	-	1	N	-	-	-	Decreased membrane fluidity via cis–trans isomerization of unsaturated fatty acids	(Hachicho et al. 2014)
Ag, Ag^+^	*P. putida*	-	75	75	Y	-	-	-	Altered expression of periplasmic and cytoskeletal proteins (FtsZ), and membrane sensors (EnvZ, GacS)	(Dong et al. 2022)
Ag, Ag^+^	*S*. Senftenberg	-	-	1	Y	-	-	-	Overexpression of silB efflux gene reducing intracellular Ag⁺	(Losasso et al. 2014)
Ag	*E. coli, K. pneumoniae, S. aureus*	-,+	-	1	Y	-	-	-	Efflux of Ag⁺ ions mediated by sil genes located on PMG101 plasmid	(Kędziora et al. 2020)
Ag	*B. subtilis*	+	-	-	Y	-	Y	-	Activation of oxidative stress defense and efflux via P-type ATPase SilP	(Gunawan et al. 2013)
Ag	*B. pumilus, E. coli, M. luteus, S. aureus*	-,+	-	1	-	-	-	-	Overproduction of exopolysaccharides leading to biofilm-mediated nanoparticle sequestration	(Khan et al. 2011)
Ag, Ag^+^	*S. aureus*	+	50	50	Y	Y -AgNO_3_	Y	-	Reduced cysteine metabolism and limited Ag⁺ influx due to PurR, TcyA, CymR regulation; enhanced oxidative stress response	(Valentin et al. 2020)
Ag	*S. aureus*	+	20	9	N	-	Y	Y—PGRE, GCN/Ag	Enhanced biofilm formation	(Hochvaldová et al. 2024)
Ag, Au	*S. aureus*	+	10	10	?	N—ATB	Y	-	Not described	(Elbehiry et al. 2019)
Au	*E. coli*	-	200	183	Y	N—ATB	Y	different AuNPs	Altered cell shape, decreased membrane permeability, and loss of flagella	(Zheng et al. 2021)
Al_2_O_3_	*E. coli*	-	-	1	Y	-	Y	-	Mutation in genes associated with membrane integrity and metal tolerance	(Zhang et al. 2018a)
ZnO	*E. coli*	-	25	4	-	-	N	-	Changes in cell morphology and upregulation of membrane-associated proteins	(Zhang et al. 2018a)
CuO	*P. aeruginosa*	-	-	1	Y	-	-	-	Upregulation of Pf1 bacteriophage-related genes promoting biofilm formation and efflux activity	(Guo et al. 2017)
GO	*P. aeruginosa*	-	160	160	Y	-	-	-	Upregulation of quorum-sensing–regulated proteases contributing to biofilm formation)	(Zhang et al. 2018b)
GO	*E. coli*	-	200	200	Y	-	-	-	Activation of envelope stress response (Cpx), increased protease secretion, and altered membrane fluidity and permeability	(Zhang and Zhang 2020)
GCN/Ag	*E. coli*	-	60	-	-	-	-	-	No adaptation observed under tested conditions	(Panáček et al. 2021)
nZVI	*P. putida*	-	10	10	N	-	-	-	Membrane rigidification via cis–trans isomerization of unsaturated fatty acids	(Kotchaplai et al. 2017)
Pt, Pd	*P. aeruginosa*	-	-	1	Y	-	-	Y—lasR	Downregulation of ROS generation and lasR-dependent biofilm pathways	(Li et al. 2021)

All parameters refer to the experimental induction and characterization of bacterial resistance toward nanoparticles. NPs—type of nanoparticles used for resistance induction; Bacteria—bacterial strain studied; Gram—Gram classification (positive “+” or negative “–”); Passage—total number of bacterial passages performed during resistance induction; Step—number of passages required to obtain a stable resistant phenotype; Gene—indicates whether genetic alterations were identified (Y = yes; N = no;—= not tested); Cross—presence of cross-resistance to other agents (e.g. antibiotics or metal salts) (Y = yes; N = no;—= not studied); Stable—stability of the resistance phenotype after passages without nanoparticles; Reversed—indicates whether the study examined possible reversal or overcoming of the induced resistance and, if so, by which approach; Mechanism—molecular or physiological adaptation identified or proposed; Author—reference to the corresponding publication.

Beyond these shared responses, additional adaptations reflect structural and physiological distinctions between Gram-negative and Gram-positive bacteria. In Gram-negatives, adaptation often involves remodeling of OM components (Zhang et al. 2018a, 2020a, Hachicho et al. 2014, Kotchaplai et al. 2017), including porins and LPS (Zhang and Zhang 2020a, Faghihzadeh et al. 2018), as well as phenotypic adjustments such as changes in fatty acid (Faghihzadeh et al. 2018) biosynthesis, altered motility (Zhang et al. 2020, Stabryla et al. 2021, Du et al. 2025), or the production of flagellin (Panáček et al. 2018, Sun et al. 2024) and pigments (Ellis et al. 2019) that interact with nanoparticles. In Gram-positives, evidence remains more limited, but studies on *S. aureus* (Hochvaldová et al. 2024), *L. monocytogenes* (Du et al. 2025), and *E. faecalis*(Cui et al. 2020) highlight enhanced biofilm formation and extracellular nanoparticle entrapment as important contributors alongside efflux. These findings indicate that, although structural differences between bacterial groups shape the specific repertoire of responses, both Gram-positive and Gram-negative species are capable of evolving diverse and multifaceted adaptive strategies to withstand nanomaterial toxicity.

### Parallels and distinctions between antibiotic and nanomaterial resistance mechanisms

**Figure 10.**
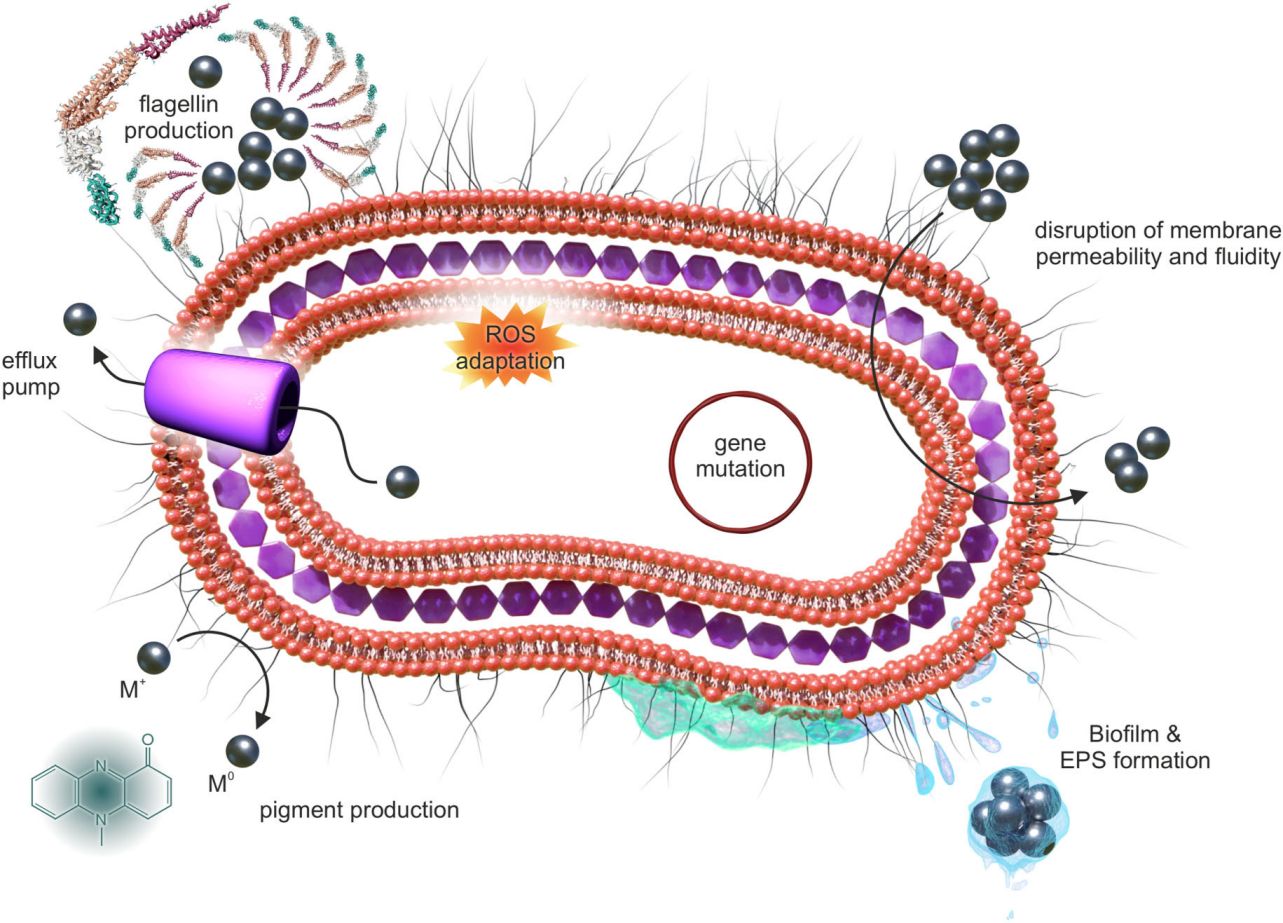
Induced mechanism of resistance towards various nanomaterials.

Understanding bacterial resistance to both traditional antibiotics and emerging nanomaterials is crucial for developing effective antimicrobial strategies. While bacteria employ several overlapping defence strategies against these agents, important distinctions arise from the differing chemical nature and modes of action of antibiotics compared with nanomaterials. Mechanisms of bacterial resistance to antibiotics are well documented, including intrinsic and acquired resistance, as reviewed by Blair and Darby et al (Blair et al. 2014, Darby et al. 2023). Adaptation to nanomaterials (Fig. 10) often manifests as an increase in the antimicrobial concentration within the cell, which is often achieved through enhanced efflux (Zhang et al. 2018b, Gunawan et al. 2013, Graves Jr. et al. 2015, Faghihzadeh et al. 2018, Kędziora et al. 2018, Valentin et al. 2020) and alterations in the bacterial envelope, resulting in decreased uptake of antimicrobial agents. While efflux systems in AgNPs and antibiotic resistance share fundamental roles in detoxifying harmful agents, the specificity, regulatory mechanisms, and adaptations diverge due to differences in the chemical nature of nanoparticles versus antibiotics. AgNPs adaptation relies more heavily on metal-ion-specific systems (e.g. *Cus, Sil*) and structural modifications, reflecting the multifaceted stress posed by nanoparticles (Silver 2003, Li and Xu 2024). On the other hand, antibiotic resistance efflux pumps, such as *AcrAB-TolC* and *MexAB-OprM*, have broader specificity, tailored to the chemical diversity of antibiotics, accommodating a range of hydrophobic antibiotics and focusing more on molecular polarity and size (Du et al. 2018, Gaurav et al. 2023).

The alterations in the bacterial envelope include reprogramming of cell wall biosynthesis, modification of OM porins (Asif and Alvi 2018, Pareek et al. 2022), reduced permeability of the cell membrane, (Zhang et al. 2018a, 2020a, Choi and Lee 2019) and morphological changes such as shape alteration and cell wall thickening (Zhang et al. 2018a,^0^), all of which inhibit the passage of nanoparticles or their ions into the bacteria, preventing them from reaching the MIC needed to inhibit bacterial growth has been described mechanisms of resistance for both nanomaterials and antibiotics (Cui et al. 2003, Yuan et al. 2013, Ghai and Ghai 2018, Ojkic et al. 2022, Zeden et al. 2023). In both cases, bacteria also develop resistance to ROS production, which plays a crucial role in the antibacterial activity of both antimicrobials (Valentin et al. 2020, Zhang et al. 2020). In *K. pneumoniae* strains that do not carry the *sil* operon, mutations in *CusS/R* regulate efflux by leading to a substantial increase in *cusA* expression (over 250-fold) of the *cusCFBA* operon, which encodes a heavy metal RND efflux pump. Decreased silver susceptibility (MICs of >512 mg/L) in these strains is achieved when these *CusS* mutations occur in conjunction with secondary mutations that disrupt the OM porin *OmpC*. These *OmpC* mutations result in a decrease in *ompC* expression levels (over 30-fold) and presumably restrict the access of silver through the OM, thereby limiting Ag⁺ uptake (Woolley et al. 2022, Li et al. 1997b).

Another key mechanism of antibiotic resistance involves the alteration of antibiotics via degrading enzymes, which modify the structure of the antibiotics, rendering them unable to bind to their bacterial targets (Egorov et al. 2018). Beyond internal cellular mechanisms, bacteria are known to produce various extracellular substances that impede contact with antimicrobial agents, thereby increasing tolerance (Flemming and Wingender 2010). While both antibiotic and MNP adaptation involve the production of specific biomolecules to counteract external threats, the types of molecules and their roles differ significantly, reflecting the distinct nature of these challenges. For antibiotic resistance, bacteria often produce highly specific enzymes, such as β-lactamases or aminoglycoside-modifying enzymes, which directly degrade or modify the antibiotic’s chemical structure, rendering it ineffective. This enzymatic action represents a targeted biochemical response to the molecular properties of antibiotics (Mora-Ochomogo and Lohans 2021). In addition to enzymatic defenses, some bacteria produce pigments as part of their strategy to resist antibiotics. For example, phenazine pigments produced by *P. aeruginosa* (Kothari et al. 2022) have been linked to increased tolerance to β-lactams, aminoglycosides, and carbapenems, often associated with elevated efflux activity and β-lactamase expression. Interestingly, similar pigment-based responses have also been observed under exposure to MNPs, (Ellis et al. 2019) where pigments can reduce oxidative stress and influence the bioavailability of toxic metal ions, thereby contributing to bacterial survival. While enzymatic mechanisms specifically target antibiotics, pigment production provides a more general protective function that can mitigate multiple types of stress, including oxidative and chemical challenges.

In contrast to antibiotics, MNPs exert multifaceted stress on bacterial cells, and several resistance mechanisms appear to be unique to nanoparticle exposure. These adaptations are generally not observed in response to conventional antibiotics and reflect the distinct physical and chemical challenges posed by nanoparticles, such as oxidative stress, ion release, and direct interactions with cellular membranes. Key nanoparticle-specific mechanisms include the synthesis of structural and extracellular components such as flagellin (Panáček et al. 2018, Stabryla et al. 2021, Sun et al. 2024), exopolysaccharides (Khan et al. 2011, Cui et al. 2020), and biofilm matrices (Zhang et al. 2018a, 2020a, Khan et al. 2011, Faghihzadeh et al. 2018, Zhang et al. 2020, Hochvaldová et al. 2024) that create physical barriers, dynamic modulation of motility, and extensive remodeling of membrane composition and cell envelope architecture to limit nanoparticle uptake and toxicity. These molecules are less specific and serves a more general protective function, addressing the multifaceted stress posed by nanoparticles, such as oxidative damage and physical interactions. This fundamental distinction highlights how bacterial adaptations are uniquely tailored to the specific nature of the threat, with antibiotic resistance often involving highly specific enzymatic degradation, while nanoparticle adaptation relies more on broader physical and chemical defenses.

Notably, the only resistance mechanisms unique to antibiotics, not observed against nanomaterials, involve mutations in DNA gyrase and topoisomerase IV, or the modification/overproduction of specific targets (Schaenzer and Wright 2020). This is likely due to these antimicrobials’ differing modes of action; antibiotics target specific cellular components, while nanomaterials have a multi-level mode of action that affects various cellular structures concurrently without requiring interaction with a specific target (Blair et al. 2014, Asif and Alvi 2018, Shaikh et al. 2019).

### Potential interventions to counteract nanoparticle adaptation

Despite the increasing number of studies describing bacterial adaptation mechanisms against metallic nanoparticles, strategies to counteract these adaptations remain poorly explored. Research on possible interventions is still limited, yet the available studies offer valuable first insights into preserving nanoparticle efficacy. Current approaches can be broadly divided into strategies that directly disrupt bacterial adaptation strategies and those that focus on modifying nanoparticle properties or delivery.

Several interventions aim to interfere with the genetic or regulatory pathways that underpin the adaptation mechanisms. For example, Li et al. proposed knocking out the *lasR* gene, a central regulator of biofilm formation in *P. aeruginosa*. Loss of *lasR* function increased ROS production and enhanced MNPs antibacterial activity (Li et al. 2021). Similarly, Sun et al. demonstrated that disrupting membrane potential with carbonyl cyanide 3-chlorophenylhydrazone or inducing overactivation of *sigma E* through heat shock sensitized *E. coli* to AgNPs. In this case, *sigma E* activation disrupted envelope homeostasis, destabilizing protective resistance states and preventing development of new protection strategies (Sun et al. 2024).

Another group of approaches focuses on inhibiting extracellular barriers or biomolecules that contribute to bacterial adaptation. Panáček et al. demonstrated that pomegranate rind extract inhibited flagellin production in *E. coli*, thereby preventing aggregation and restoring nanoparticle susceptibility (Panáček et al. 2018). The same extract also suppressed biofilm formation and aggregation in *S. aureus*, which otherwise reduces nanoparticle efficacy (Hochvaldová et al. 2024). In a different model, Cui et al. reported that co-treatment of *E. faecalis* with AgNPs and simvastatin disrupted EPS entrapment, reinstating nanoparticle penetration and bactericidal activity (Cui et al. 2020). Together, these findings illustrate that targeting extracellular defensive factors, such as flagellin, biofilm, or EPS represents a promising route for enhancing nanoparticle performance.

Other interventions emphasize modifying nanoparticle characteristics or delivery methods to overcome bacterial defenses. Preventing nanoparticle aggregation is a key concern, as aggregation reduces surface reactivity and antimicrobial activity. Hochvaldova et al. showed that covalently binding nanoparticles to cyanographene sheets effectively prevented aggregation and maintained antibacterial properties (Hochvaldová et al. 2024). Adjusting nanoparticle physicochemical parameters has also been proposed: Zheng et al. observed that reduced susceptibility to AuNPs in *E. coli* was size-specific and could be overcome by optimizing nanoparticle size (Zheng et al. 2021). Another innovative strategy exploits enhanced bacterial uptake through the so-called “Trojan horse” effect, where silver is combined with nutrients to promote active bacterial uptake of the toxic agent, thereby bypassing surface defenses (Panáček et al. 2018, Zharkova et al. 2021, Hochvaldová et al. 2024, Li and Xu 2024, Sun et al. 2024).

There are notable parallels between interventions against nanoparticle adaptations and those developed for antibiotics. In antibiotic-resistant strains, treatment often involves switching to alternative drugs, combining antibiotics with resistance inhibitors (e.g. β-lactamase inhibitors), or employing agents that increase membrane permeability (Annunziato 2019). Analogously, nanomaterial-based strategies often combine MNPs with inhibitors targeting bacterial adaptation mechanisms (e.g. inhibitors of flagellin, biofilm, or efflux pumps). However, some approaches are unique to nanomaterials, such as immobilizing nanoparticles onto substrates to prevent aggregation or exploiting the Trojan horse effect to enhance uptake. These distinctions highlight that while conceptual similarities exist, nanomaterial adaptation also requires tailored interventions that account for their distinct modes of antibacterial action.

Despite these advances, the field remains in its infancy. Most studies to date have focused on characterizing adaptation mechanisms rather than systematically developing counter-strategies. The few available examples underscore the feasibility of overcoming bacterial adaptation through either targeting or nanoparticle modification, yet comprehensive frameworks are still lacking. Future research should prioritize systematic exploration of these interventions, assess their generalizability across bacterial species and nanoparticle types, and evaluate risks such as cross-resistance or unintended ecological effects.

### Induction of bacterial adaptation across studies

One of the most pressing challenges in the study of bacterial adaptation to antimicrobial nanomaterials is the lack of methodological consistency across published studies. While the field is rapidly growing and holds great promise, the variability in experimental approaches currently hinders reliable comparisons and makes it difficult to draw robust conclusions about the likelihood and dynamics of microbial adaptation.

In most studies on nanomaterial-bacteria interactions, the term resistance is commonly used. However, this terminology is problematic. In the context of antibiotics, resistance and sensitivity are clinically defined concepts that rely on established breakpoints, which allow clear classification of bacterial susceptibility. Such breakpoints do not exist for nanomaterials, and the mechanisms underlying bacterial responses are often complex, multifactorial, and non-specific. Therefore, the direct application of the term resistance to nanomaterials is debated and can be misleading. In this review, we instead use the terms adaptation, reduced susceptibility, or non-susceptibility to describe bacterial responses that lead to decreased effectiveness of antimicrobial nanomaterials.

A key source of inconsistency lies in the duration of bacterial induction to sublethal concentrations of the nanoparticles. Reported induction times range widely, from as few as one passage to as many as 200. Equally variable are the methods used to assess the “resistance” development. In some cases, minimum inhibitory concentrations (MICs) were determined after each cultivation step, (Zhang et al. 2018a, Panáček et al. 2018, Ellis et al. 2019) allowing for precise tracking of adaptation development. In most studies, however, researchers evaluated MICs only after predetermined intervals, such as the 10th (Elbehiry et al. 2019), 17th (Elbehiry et al. 2019), 50th (Valentin et al. 2020), 100th (Graves Jr. et al. 2015), 160th (Zhang et al. 2018c), or 200th (Zhang and Zhang 2020b,^0^) passage. Such methodological variation makes direct comparison difficult and creates uncertainty in interpreting results.

This inconsistency has important consequences. For example, adaptation may appear undetectable after 10 passages in one study, whereas another may report clear adaptation only after 200 passages. This raises the critical question. Is the increased MIC evidence of true resilience, or simply a result of insufficient induction time? The lack of standardization leaves this distinction unresolved. Several studies illustrate this challenge. Mao et al. reported that lysine-derived carbonized nanogels appeared to suppress the evolution of new adaptation mechanisms, but their experiments covered only 20 passages, likely too few to exclude long-term adaptation (Mao et al. 2022). Similarly, Panáček et al. observed no decrease of bacterial susceptibility to silver nanoparticles immobilized on cyanographene surfaces after 60 passages, yet this still leaves open whether bacterial adaptation might emerge over longer experimental durations (Panáček et al. 2021). These examples underscore how methodological choices can fundamentally shape conclusions.

Taken together, the evidence suggests that the likelihood of detecting reduced susceptibility increases with the number of induction cycles. Thus, there is an urgent need for standardized methodologies that define the minimal number of cycles required to make reliable claims about the inability of a material to induce some bacterial adaptations. Such protocols would ensure consistency, comparability, and greater confidence in evaluating antibacterial nanomaterial resilience. Looking ahead, future research should prioritize the development of these unified induction and assessment protocols. In parallel, studies should investigate whether nanoparticle adaptations can confer cross-resistance to conventional antibiotics, a largely unexplored but highly relevant issue. Finally, beyond standardization, research must explore strategies to prevent the formation of adaptive mechanisms altogether, including targeting specific genetic pathways or employing combination therapies. Establishing methodological clarity will provide a stronger foundation for such innovations, ensuring progress in both understanding and overcoming nanoparticle adaptation mechanisms.

## Concluding remarks

This review highlights that bacteria are capable of building adaptive strategies even to nanoparticles. For a long time, the absence of confirmed adaptation mechanisms to nanoparticles, especially AgNPs, was attributed to their multimodal antibacterial effects—acting simultaneously on multiple levels and through various mechanisms. However, recent evidence has demonstrated that bacteria can indeed develop defense mechanism even against these antibacterial agents, which challenges the previous assumptions about the robustness of nanoparticle-based antimicrobials. Common adaptation mechanisms, such as efflux pumps, biofilm formation, and membrane alterations, parallel those observed with antibiotics, yet unique mechanisms specific to nanoparticles have also emerged. For example, bacteria exhibit novel adaptive strategies related to nanoparticle aggregation and surface interactions, which have not been observed with traditional antibiotics. These adaptations include bacterial motility, corona formation and production of pigments and other substances, all of which contribute to nanoparticle adaptation. This underscores bacteria’s extraordinary adaptability and capacity for developing entirely original adaptation strategies. To overcome and prevent bacterial adaptation to nanoparticles, it is essential to thoroughly understand these mechanisms and develop emerging strategies tailored to these unique responses.

## Data Availability

No new data were generated or analysed in support of this research.
